# Hydrodynamic Noise of Pulsating Jets through Bileaflet Mechanical Mitral Valve

**DOI:** 10.1155/2020/1024096

**Published:** 2020-05-30

**Authors:** Vladimir Voskoboinick, Oleksandr Voskoboinyk, Oleg Chertov, Andrey Voskoboinick, Lidiia Tereshchenko

**Affiliations:** ^1^Institute of Hydromechanics of NAS of Ukraine, Kyiv 03057, Ukraine; ^2^NTUU Igor Sikorsky Kyiv Polytechnic Institute, Kyiv 03057, Ukraine

## Abstract

Experimental research results of hydrodynamic noise of pulsating flow through a bileaflet mechanical mitral valve are presented. The pulsating flow of pure water corresponds to the diastolic mode of the cardiac rhythm heart. The valve was located between the model of the left atrium and the model of the left ventricle of the heart. A coordinate device, on which a block of miniature sensors of absolute pressure and pressure fluctuations was installed, was located inside the model of the left ventricle. It is found that the hydrodynamic noise of the pulsating side jet of the semiclosed valve is higher than for the open valve. The pressure fluctuation levels gradually decrease with the removal from the mitral valve. It is established that at the second harmonic of the pulsating flow frequency, the spectral levels of the hydrodynamic noise of the semiclosed bileaflet mechanical mitral valve are almost 5 times higher than the open valve. With the removal from the mitral valve, spectral levels of hydrodynamic noise are decreased, especially strongly at the frequency of the pulsating water flow and its higher harmonics.

## 1. Introduction

The heart is a vital hollow muscular-fibrous organ located in the thorax and providing blood flow through the vessels. This is a kind of muscle pump that works on the principle of suction-pushing blood. The human heart is divided by diaphragms into four separate chambers: two atria (left and right) and two ventricles (also left and right). The functions of each of them are different. Inside each of the atria, blood entering the heart is accumulated and, having reached a certain volume, is pushed into the corresponding ventricles. The ventricles drive blood into the arteries, through which it moves throughout the body. The unidirectional movement of blood is ensured by the well-coordinated work of the valvular apparatus of the heart, consisting of the mitral, tricuspid, aortic, and pulmonary valves, which are opened and closed at the right moment, preventing the blood from being regurgitated. The first and third valves are located in the left ventricle, and the second and fourth valves are located in the right ventricle of the heart.

The heart pumps about five to six liters of blood in a minute. This volume is somewhat decreased at rest and when a person performs physical exercise, on the contrary, it is increased. Together with blood vessels, the heart forms the cardiovascular system, which has two circles of circulation: large (systemic) and small (pulmonary). Blood first enters in the aorta from the left ventricle of the heart and then it moves through large and small diameter arteries. Then blood moves through the arterioles to the capillaries, where it donates oxygen and a number of other nutrients necessary for the body and takes carbon dioxide and waste metabolic products. Hence, the blood from the artery (oxygenated blood flows from the heart) becomes venous blood and flows back to the heart. The venous blood flows first through the venules, then through the small veins and large venous trunks. The venous blood enters inside the right atrium along the inferior and superior vena cava, closing the systemic circulation (large circle). The blood is again enriched with oxygen in the lungs, where it flows from the right heart through the pulmonary valve into the pulmonary arteries, which form the pulmonary circulation. Oxygenated blood fills the left atrium and through the mitral valve enters the left ventricle of the heart. The mitral (bicuspid or bileaflet) valve is located between the left atrium and the ventricle and consists of two leaflets. When it is opened, blood flows through an atrioventricular orifice into the left ventricle from the left atrium. During systole (i.e., contraction) of the left ventricle, the valve is closed so that blood does not flow back into the atrium, but is pushed through the aortic valve into the aorta and the vessels of the systemic circulation. Heart valves consist of thin, flexible leaflets that are opened and closed in response to changes in blood pressure between the respective atria and ventricles. As a result of various diseases and pathologies, the leaflets are damaged and interfere with the normal functioning of the heart and the entire cardiovascular system. As a result, it is necessary to apply therapeutic and surgical measures to eliminate such injuries until the replacement of natural heart valves with prostheses. About 300 thousand prosthetic heart valves are annually implanted in the world, and it is estimated [1] that the number can triple in 2050.

**Figure 1:**
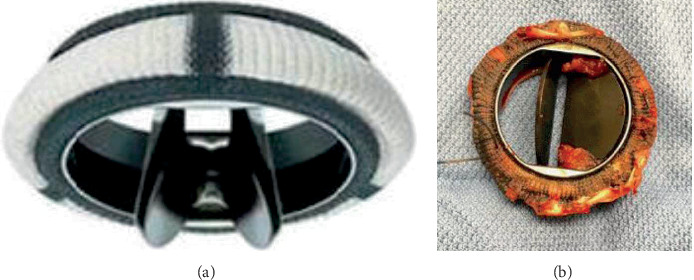
Bileaflet mechanical heart valve: open (a) and semiclosed by trombi (b).

In recent years, a large number of heart valve prostheses of various forms and principles have been developed and put into practice, which are grouped into three groups: bioprostheses, mechanical, and transcatheter valves [2–5]. Most valves are manufactured by world-renowned firms such as Edwards Lifesciences, Medtronic, St. Jude Medical, Sorin Group, and Boston Scientific. The bileaflet mechanical heart valves are most common (more than 50% of prosthetic valves). These valves consist of two semilunar leaflets (Figure 1(a)), which are attached to a rigid stitched ring by means of small hinges. The opening angle of the valves relative to the plane of the ring is ranged from 75 to 90°. An open valve has three openings: a small slit-like central opening between two open leaflets and two larger semicircular openings on the sides of the valve. The valves are mainly made of pyrolytic carbon, because it has a sufficiently high thromboresistance. Despite the wide clinical application, the functions of these valves are far from perfect. The main disadvantages that distinguish them from the ideal mechanical heart valve are the destruction of erythrocytes and the formation of hemolysis, that is, the destruction of red blood cells, as well as thromboembolism resulting from the formation of thrombi on the streamlined surface of mechanical valves (Figure 1(b)). At the same time, patients with mechanical heart valves should use anticoagulants throughout their lives to counteract thromboembolic complications.

## 2. Materials and Methods

Since mechanical valves have nonphysiological geometry, the flow of blood through them is significantly different from natural conditions. Indeed, the hemodynamics of bileaflet mechanical heart prostheses is significantly different from natural valves, since they have three orifices of different sizes. This forms a localized high-velocity gradient through a smaller central opening. The leaflets of the valve are a barrier to the blood flow through the valve, which, along with the high velocity of the jets between the leaflets, causes increased shear stresses, which lead to the destruction of erythrocytes and other blood corpuscles [6–8]. In addition, there is a small technological gap between the leaflets which are fixed in the ringed valve body by means of hinges, and the ring itself. The reverse blood flow is rushed with a sufficiently high velocity in this gap, generating large shear stresses, that increases the risk of damage to the blood and the formation of thrombi.

Optimization of the design of mechanical valves is achieved using computational and experimental methods [9–11] to improve the velocity profiles and minimize the complications that are caused by prosthetics. Modern numerical simulation methods such as DNS, URANS, LES, and DES are used for calculations [12–14]. Hemodynamic and hydrodynamic tests of heart valves are an important step for conducting preclinical research of a new device. The main indicators of the effectiveness of such devices are the velocity profiles, velocity and pressure gradients, shear and Reynolds stresses, convective velocities, and directions of motion of vortex structures that are formed by a heart valve [15–17]. Studies of the blood flow characteristics inside the heart and the operation conditions of mechanical valves are performed both by noninvasive measurement methods (in vivo) and with help ventricular and atrial models in laboratory conditions (in vitro). Research by noninvasive methods is carried out mainly on the rather complex equipment of Doppler echocardiography and magnetic resonance imaging.

But this modern equipment has a serious disadvantage due to insufficient temporal and spatial resolution. Therefore, the fine structure of blood flow through the mechanical heart valves is investigated in the laboratory with the help of miniature sensors and complexes for detecting the movement of labelled particles with an increased spatial and temporal resolution [18, 19]. Several complex experimental setups that were used for hemodynamic and hydrodynamic studies have been described in the literature [20–22].

Diagnostics of the operation of mechanical valves in vivo are carried out using diagnostic complexes of Doppler echocardiography, magnetic resonance imaging, electrocardiography, ultrasound tomographic velocity measurement, phonocardiography, seismocardiography, and a number of other techniques and devices [23–26]. This equipment uses various principles and mechanisms for recording hemodynamics inside the heart and in general throughout the entire cardiovascular system. Each type of diagnosis has its advantages, as well as disadvantages, which are quite well covered in the scientific literature [27–29]. However, there is the problem of creation of the effective, inexpensive, and miniature diagnostic equipment for the registration of the thrombus formation on the leaflets of mechanical heart valves, since thrombi prevent the valve opening. If one of the leaflets is closed, as shown in Figure 1(b), it is urgently necessary to take appropriate measures to replace the valve or eliminate thrombi. It is desirable to create such a device that patients could use this device at home without receiving a special medical education.

The goal of our research is to develop principles and methods for vibro-hydroacoustic diagnostics of the operation of a bileaflet mechanical heart valve, as well as to study the features of the transformation of hydrodynamic noise and vibrations that are generated inside the left ventricle and atrium models and transmitted to the surface of the laboratory bench.

### 2.1. Experimental Setup

**Figure 2:**
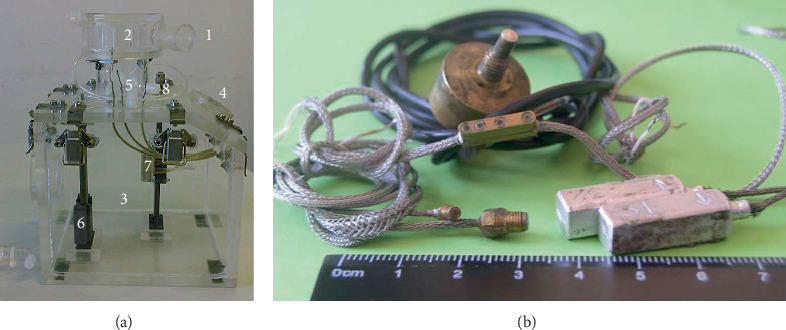
Experimental bench (a), piezoresistive and piezoceramic sensors (b).

Experimental research of the pure water flow, the density of which is close to the density of blood, and the kinematic viscosity coefficient is (4-5) times lower than that of blood, in the diastole, a regime was carried out in the microlaboratory of Politecnico di Milano (Italy). The bileaflet mechanical heart valve with a diameter of *d* = 25 mm from Sorin biomedica (Italy), which is shown in Figure 1(a), was used in this research. The valve was located between the model of the left atrium and the model of the left ventricle of the heart and worked as a mitral valve. The models of an atrium and a ventricle were made of organic glass and are shown in Figure 2(a).

Water was entering inside the atrium model (2) and the left ventricular model (3) through the inlet fitting (1). A device (5) was made between the atrium and the ventricle, where the bileaflet mechanical heart valve was installed. Water from the experimental bench flowed out through the outlet fitting (4). Inside the model of the left ventricle, there was a coordinate device (6) on which a block of miniature absolute pressure sensors and pressure fluctuation sensors (7) was installed. The coordinate device allowed the sensors to be moved downstream from the bileaflet valve along the direction of the jets that flowed out from an open or semiclosed valve. Vibrations on the surface of the experimental bench, absolute pressure, and pressure fluctuations inside it were recorded using single-component piezoceramic accelerometers, as well as miniature piezoresistive and piezoceramic absolute pressure sensors and pressure fluctuation sensors (Figure 2(b)). These sensors were developed and manufactured at the Institute of Hydromechanics of the National Academy of Sciences of Ukraine [30, 31].

**Figure 3:**
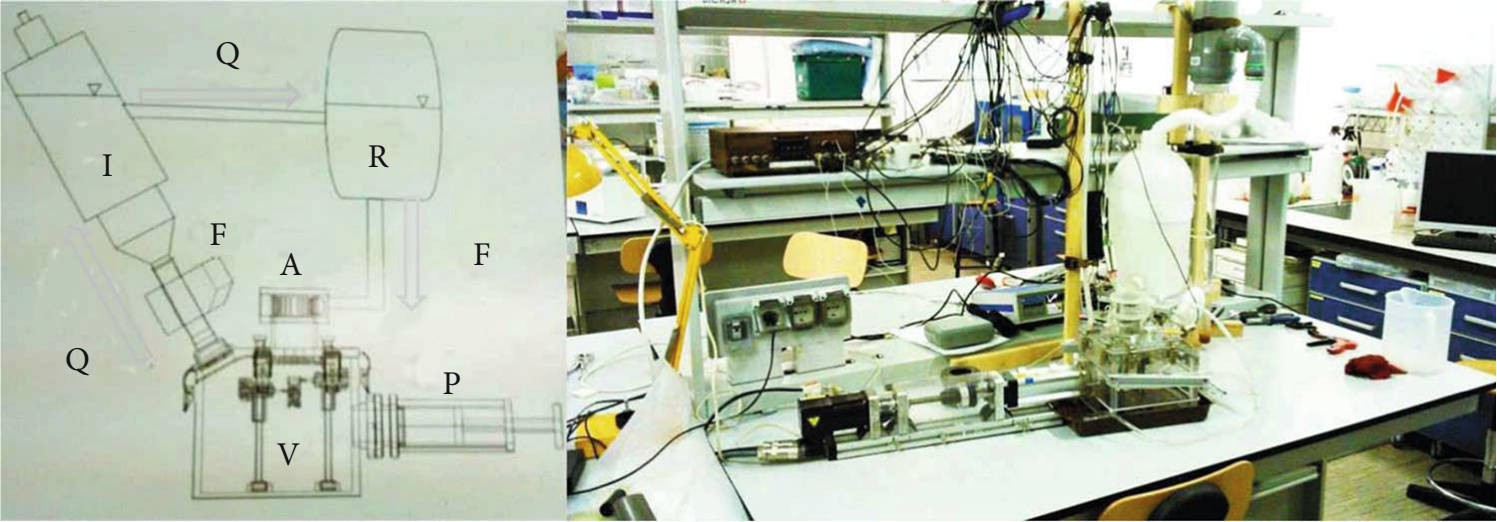
Scheme and photography of the experimental stand.

To conduct hydroacoustic diagnostics of the operation of the bileaflet mechanical mitral valve, a special experimental stand was created [32, 33]. The scheme and photograph of the stand are shown in Figure 3. The impulse pump was pumping water through the mitral valve from the reservoir (R) to the impedance tank (I). The impulse pump was controlled by a computer using a specially developed program, which made it possible to create a pulsating water supply of a certain amplitude, period, and form. In our studies, the pulse form of water movement through the mitral valve corresponded to the cardiac cycle of diastole.

The impedance tank provided the lower threshold of the arterial pressure of the cardiac cycle. An ultrasonic flowmeter (F) was installed at the inlet or outlet of the experimental bench. Thus, the computer-controlled operation of the impulse pump made it possible to accurately and stably control the pulse form of the water supply through the mitral valve (diastole regime), the pulse period (cardiac pulse), and the water flow rate. In studies, the frequency of the pulsating water supply was 1 Hz or 60 beats per minute, and the average flow rate through the open or semiclosed mitral valve varied from 3 l/min to 6 l/min.

**Figure 4:**
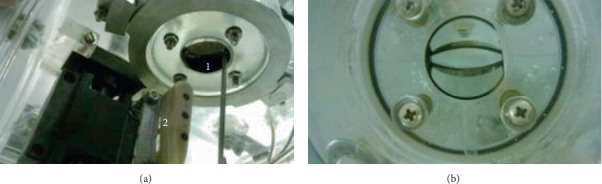
Location of the pressure sensors downstream of the open bileaflet valve.

Studies of the hydrodynamic noise of water jets, which flowed out from the mitral valve into the model of the left ventricular chamber, were carried out using a block of pressure fluctuation sensors [34, 35]. These sensors were located inside the experimental bench on the coordinate device and were located downstream of the valve, as shown in Figure 4. Miniature piezoceramic pressure fluctuation sensors (diameter of the sensitive surface of 1.3 mm) were installed in the well-streamlined block of pressure sensors at a fixed distance from each other (Figure 4(a)). Holes with a diameter of 0.5 mm were made here, through which the absolute pressure was measured by piezoresistive pressure sensors. The sensor block through the coordinate device was moved along the studied jets, which flowed out through an open or semiclosed valve. Thus, the sensors recorded the hydroacoustic noise of the near field of the jets (Figure 4(b)). The mounting structure of the mitral valve made it possible to turn the valve around its axis. Consequently, the sensors recorded the noise of either a side jet or a central jet of the bileaflet valve.

The electrical signals of the sensors were amplified by low-noise amplifiers and were given to personal computers using multichannel analog-to-digital converters. Processing and analysis of experimental results were carried out using probability theory and mathematical statistics.

According to the developed program and research methodology, the vibroacoustic diagnostics of the experimental bench was originally conducted. Sources of extraneous vibrations and noises were established, and measures were taken to eliminate them or to reduce noise levels. The sensors were periodically checked; the measurement errors of the integral and spectral characteristics of the research parameters were determined. This allowed to receive experimental results with acceptable accuracy and good repeatability.

The measurement error of averaged and integral values of the pressure and vibration fields did not exceed 10% (95% reliability). The measurement error of the velocity rate is no more than 3%. The measurement error of the spectral characteristics of the fields of velocity, pressure fluctuations, and accelerations is no more than 2 dB in the frequency range from 0.01 Hz to 2 kHz with a confidence probability of 0.95 or 2*σ*.

## 3. Results and Discussion

**Figure 5:**
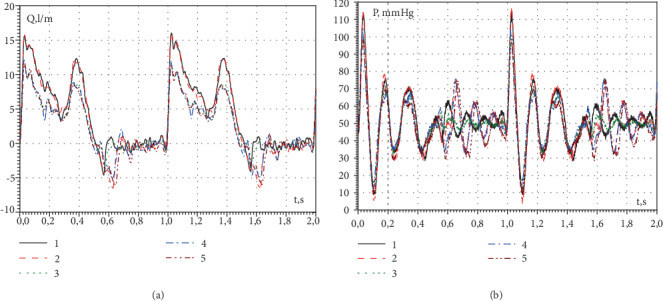
The pulsating water supply (a) and the pressure changes (b) inside the ventricular model.

The impulse pump pumped water through the mitral valve in accordance with the diastole mode of the operation heart. In this mode, two pulsed blood supply occur through the mitral valve. The first impulse is formed by the expansion of the left ventricle of the heart (wave E), and the second is formed by the contraction of the left atrium (wave A). Between these impulses, there is a diastase time interval during which the volume of the ventricle is constant [36, 37]. The form of the curves of the pulsating water supply by the pump through the mitral valve in the form of water rate, which was recorded by an ultrasonic flowmeter, is shown in Figure 5(a). Here, the flow rate measured in l/min is shown as a function of the impulse time. Curves 1 and 2 simulate the blood flow through the left ventricle of a small person (71% of the pump power), and curves 3-5 simulate the blood flow through the left ventricle of a teenager (50%). Curves 1 and 3 were measured to supply clean water under the operating conditions of a semiclosed valve, and curves 2, 4, and 5 were measured to supply water through an open mitral valve. The first, higher impulse of water rate, corresponds to wave E, and the second impulse corresponds to wave A of diastole. The pressure changes inside the left ventricular model are shown in Figure 5(b) in the working conditions of open and semiclosed mitral valve in the process of diastole. Here, the numbers of the curves correspond to those shown in Figure 5(a).

**Figure 6:**
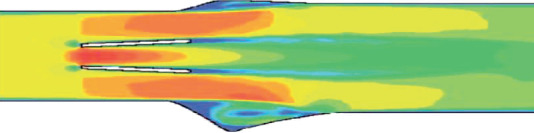
Scheme of fluid flow through an open mitral valve.

The liquid flow through the open mitral valve was divided into three jets—the central and two side jets, which are schematically shown in Figure 6. When one of the leaflets of the mechanical bileaflet valve of the heart is closed, then the blood flows only through the open leaflet. In this case, the flow velocity in the side jet of the open valve leaflet and partially in the central jet increases. This situation occurs when one of the valve leaflets is closed by thrombi, and only the open leaflet of the mechanical heart valve is working.

**Figure 7:**
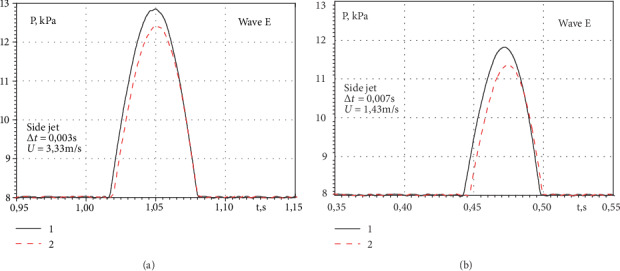
Pressure fluctuations in the near wake of the side jet of the open and semiclosed mitral valve for the operating conditions of the pump 50% (a) and 71% (b).

The pressure fluctuation dependences, measured in the near wake of the side jet, under the conditions of open and semiclosed mitral heart valve to simulate diastole of a teenager and a small person are shown in Figure 7. In these figures, curve 1 corresponds to the operating conditions of the open mitral valve, and curve 2 corresponds to the operating conditions of the semiclosed valve. In accordance with the above results, the intensity of pulsating pressure fluctuations or pulsating hydrodynamic noise of the side jet in the near wake of the valve at a distance d from it is almost 1.5 times higher for a small person and in the operating conditions of the semiclosed bileaflet mechanical mitral valve than in the conditions of the open heart valve.

**Figure 8:**
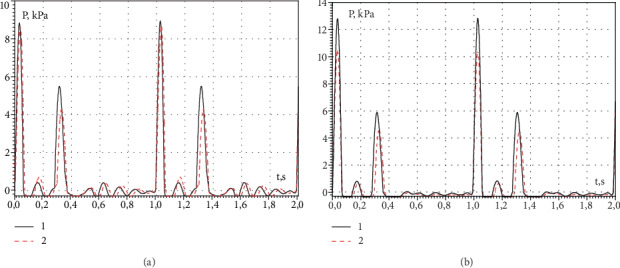
Pressure fluctuations of the wave E in the near wake of the side jet of the semiclosed (a) and open (b) mitral valve for the operating conditions of the pump 71%.

Short-term statistical processing of the research results of pulsating water supply through the mitral heart valve allowed us to separate the impulses of wave E and wave A of diastole. In Figure 8 is shown the averaged impulses of the wave E of the side jet of the semiclosed and open valve, measured by two pressure fluctuation sensors, which are spaced 0.4 d from each other. Curve 1 was measured by the sensor at a distance of 1.2 d downstream of the mitral valve, and curve 2 was measured by the sensor at a distance of 1.6 d from the valve. The time delay between the recordings of the impulse maxima was 0.003 s and 0.007 s for the operating conditions of the semiclosed and open valve and the modelling of the heart operation of the small person. At the same time, the maximum of impulses was first observed in signals that were registered sensors located closer to the mitral valve. This made it possible to determine the maximum transfer velocity [38–40] of the wave E of diastole, which was almost 1.4 m/s for the operating conditions of the open mitral valve of the heart and 3.3 m/s for the operating conditions of the semiclosed heart valve of the small person.

**Figure 9:**
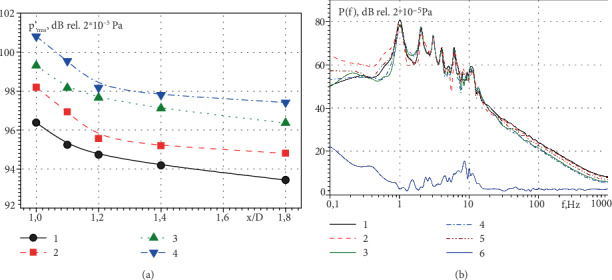
RMS of the pressure fluctuations (a) and their spectral levels (b).

Changes of the root-mean-square (RMS) values of the pressure fluctuations along a side jet that pulses with a frequency of 1 Hz are shown in Figure 9(a) for the operating conditions of the mitral heart valves of the teenager and the small person. Here, curves 1 and 2 were measured for the operating conditions of 50% of the pump power, and curves 3 and 4—71% of the pump power. Curves 1 and 3 were measured for open mitral valve and curves 2 and 4 are for semiclosed valve. The integral characteristics of the hydrodynamic noise were measured in the wake of the bileaflet valve when the sensor block was removed along the side jet. The RMS values of pressure fluctuations in the wake of the side jet of the mitral valve of a small person are more than 1.5 times higher than that of a teenager practically throughout the jet. During the pulsating flow of the semiclosed valve, the hydrodynamic noise of the side jet is higher than when the valve is open. This correlates with studies for a stationary water flow through the mitral valve [33, 35, 37]. The pressure fluctuation levels gradually are decreased when sensors are moved away from the mitral valve.

The spectral levels of the pressure fluctuations along a pulsating side jet that was flowed out from the semiclosed mitral valve, when simulating diastole of the teenager's heart, are shown in Figure 9(b). Here, curve 1 was measured at a distance d from the mitral valve, curve 2 was measured at a distance of 1.1 d, curve 3 was measured at a distance of 1.2 d, curve 4 was measured at a distance of 1.4 d, curve 5 was measured at a distance of 1.8 d, and curve 6 was ambient noise. According to research results, in the frequency range up to 20 Hz, the dynamic measurement range exceeds 30 dB. When distance from the mitral valve is increased, the spectral levels of hydrodynamic noise are decreased, especially strongly at the frequency of pulsating water flow and its higher harmonics. Harmonics of higher orders are formed due to the nonlinear interaction between the vortex and jet flows that flow through the heart valve and their interaction with the components of the valve and heart.

**Figure 10:**
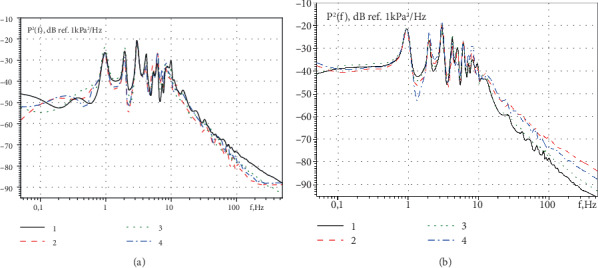
The spectral power densities of the pressure fluctuations in the near wake of the central jet of the mitral valve for the operating conditions of the pump 50% (a) and 71% (b).

The spectral power densities of the pressure fluctuations measured in the near wake of the mitral valve along the central jet that pulses with a frequency of 1 Hz are shown in Figure 10 for the model conditions of the diastole of the teenager and the small person. Here, curves 1 and 2 were measured by a pressure transducer that is removed from the mitral valve by a distance d, and curves 3 and 4 were measured by a pressure transducer that is removed from the mitral valve by a distance of 1.4 d. Curves 1 and 3 were measured for the operating conditions of the semiclosed valve, and curves 2 and 4 were measured for the operating conditions of the open valve. In the spectral dependences, discrete peaks were observed at the frequency of the pulsating flow and its higher harmonics. The spectral levels of pressure fluctuations when was simulated diastole of the small person are higher than when was simulated diastole of the heart rhythm of the teenager in the entire frequency range of the research.

**Figure 11:**
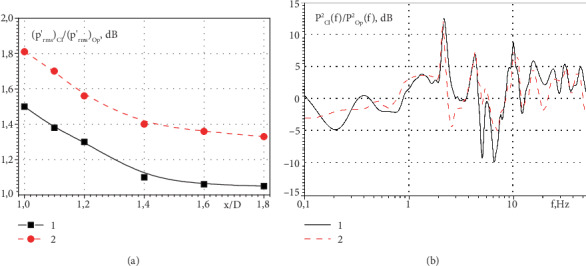
The ratios of RMS values of the pressure fluctuations (a) and the spectral levels of the hydrodynamic noise (b) in the near wake of the side jet of the mitral heart valve.

The research results showed that the RMS values of the pressure fluctuations of the hydrodynamic noise of the side and central jets, which are move from the mitral heart valve, are larger for a small person than for a teenager, which is illustrated in Figure 9(a) and Figure 11(a). Figure 11(a) shows the ratio of the RMS values of the pressure fluctuations along the side jet downstream of the semiclosed and open valve.

Curve 1 was measured for the operating conditions of the mitral heart valve of the teenager, and curve 2 was measured for the operating conditions of the mitral heart valve of the small person. The greatest difference of the pressure fluctuations was observed in the near wake of the valve, and with an increase of the distance from the valve, the ratio of RMS pressure fluctuations gradually decreases.

The ratios of the spectral power densities of the pressure fluctuations in the near wake of the side jet of the semiclosed and open valves for the operating conditions of the heart valve of the small person are shown in Figure 11(b). Curve 1 was measured by a sensor that was removed at a distance d from the mitral valve, and curve 2 was measured by a sensor that was removed at a distance of 1.4 d from this valve. The highest ratio of the pressure fluctuation levels (almost 5 times) was observed at the second harmonic of the frequency of the pulsating water flow. Along with this, when the distance from the mitral heart valve is increased, the ratios of the spectral components of the hydrodynamic noise of the semiclosed and open valve are decreased.

## 4. Conclusions

1. It was found that the intensity of the hydrodynamic noise of the pulsating side jet in the near wake of the semiclosed bileaflet mechanical mitral heart valve is almost 1.5 times higher than the open valve. The levels of the pressure fluctuations of the hydrodynamic noise gradually decrease with moving away from the mitral valve
2. It was established that the maximum transfer velocity of the wave E of the diastole inside the left ventricular model for operating conditions of the open mitral valve of the small person is almost 1.4 m/s and about 3.3 m/s for the operating conditions of the semiclosed heart valve
3. It was registered that the spectral levels of the pressure fluctuations when is simulated diastole of the heart valve of the small person are higher than when is simulated diastole of the heart valve of the teenager in the entire frequency range of the research (from 0.1 Hz to 1 kHz). In the spectral dependences of the hydrodynamic noise, discrete peaks were observed at the frequency of the pulsating water flow and at its higher harmonics. With an increase of the distance from the mitral valve, the spectral levels of hydrodynamic noise were decreased, especially strongly at the frequency of the pulsating flow and its higher harmonics.

## Data Availability

All data included in this study are available upon request by contact with the corresponding authors.
